# Endogenous bufadienolides, mineralocorticoid receptor antagonists and fibrosis in chronic kidney disease

**DOI:** 10.3389/fphar.2024.1431216

**Published:** 2024-09-04

**Authors:** Mai Rosenberg, Alexei Y. Bagrov

**Affiliations:** ^1^ Department of Internal Medicine, Institute of Clinical Medicine, University of Tartu, Tartu, Estonia; ^2^ Padakonn Pharma, Narva, Estonia

**Keywords:** chronic kidney disease, Na/K-ATPase, marinobufagenin, canrenone, mineralocorticoid receptor antagonists, Fli1, TGF-beta, collagen-1

## Abstract

Every year millions die prematurely of complications related to chronic kidney disease (CKD). Main causes of death are connected with cardiovascular (CV) complications. There is no cure for CKD although current treatment can slow the progression of the disease if diagnosed early. Fortunately, last decades have witnessed an accelerating pace of discovery regarding the cellular and molecular basis for CKD and CV disease. Novel biomarkers, including amino-terminal type III procollagen peptide (PIIINP), carboxy-terminal type I procollagen peptide (PICP), FGF23, marinobufagenin, and several miRNAs, show promise for early detection and risk stratification. In this review, we provide an overview of recent advances in the “fibrotic concept” of the etiology and pathogenesis of CKD which involves system consisting of Na/K-ATPase and its endogenous ligands including marinobufagenin which inhibits Fli1 and stimulates synthesis of collagen-1 in the vasculature. A novel treatment of CKD already involves the use of mineralocorticoid receptor antagonists capable of impairing marinobufagenin-Na/K-ATPase interactions.

## 1 Introduction

Chronic kidney disease (CKD) is a major public health problem and around 10% of the adult population have some form of kidney damage. Patients with CKD have an unacceptably high mortality rate, and their cardiovascular (CV) mortality is several times higher in patients on dialysis than in the rest of population (Jankowski et al., 2021). Cardiomyopathy in CKD have been investigated extensively during last several decades. Experimental studies revealed high variability in results, especially regarding cardiac hypertrophy and systolic function (Soppert et al., 2022). In humans, common clinical features and biomarkers are present in uremic cardiomyopathy. Parameters of kidney and cardiac damage have been associated with increased CV risk in patients with CKD (Junho et al., 2023). Characterization of the pathophysiological factors of increased cardiorenal risk is needed for the rational design of novel clinical trials (Little et al., 2023; Dobre et al., 2024).

## 2 Cardiotonic steroids

In addition to known biomarkers, there is also an opportunity to investigate novel biomarkers/factors related to the pathogenesis of CKD and CV disease. These would be cardiotonic steroids (CTS), including one of them, marinobufagenin (MBG), which is an important factor (Bagrov et al., 2009). In this review, we focus on these factors as well as on underlying pathophysiological mechanisms little discussed in literature.

CTS inhibits the Na/K-ATPase (NKA) and regulates the monovalent ions balance and cell homeostasis. The physiological activity of the NKA is determined by the maintenance of the ion gradient, a key factor in the reabsorption of sodium and potassium ions which creates a balance of osmotic pressure in cells and tissues, and makes it possible to create and maintain the membrane potential (Goto et al., 1992; Bagrov et al., 2009). By binding to the NKA, CTS can affect cell growth and differentiation, apoptosis, and proliferation (Orlov et al., 2013; Orlov et al., 2020). An important effect of CTS is their ability to function as pro-fibrotic factors i.e., to initiate intracellular signaling cascades leading to a loss of elasticity and vascular fibrosis (Elkareh et al., 2007; Elkareh et al., 2009). One of the mechanisms underlying the pro-fibrotic effect of MBG is the altered activity of Fli1, a nuclear transcription factor and a negative regulator of collagen-1 synthesis (Elkareh et al., 2009; Nikitina et al., 2011).

## 3 Fli1 signaling

**FIGURE 1:**
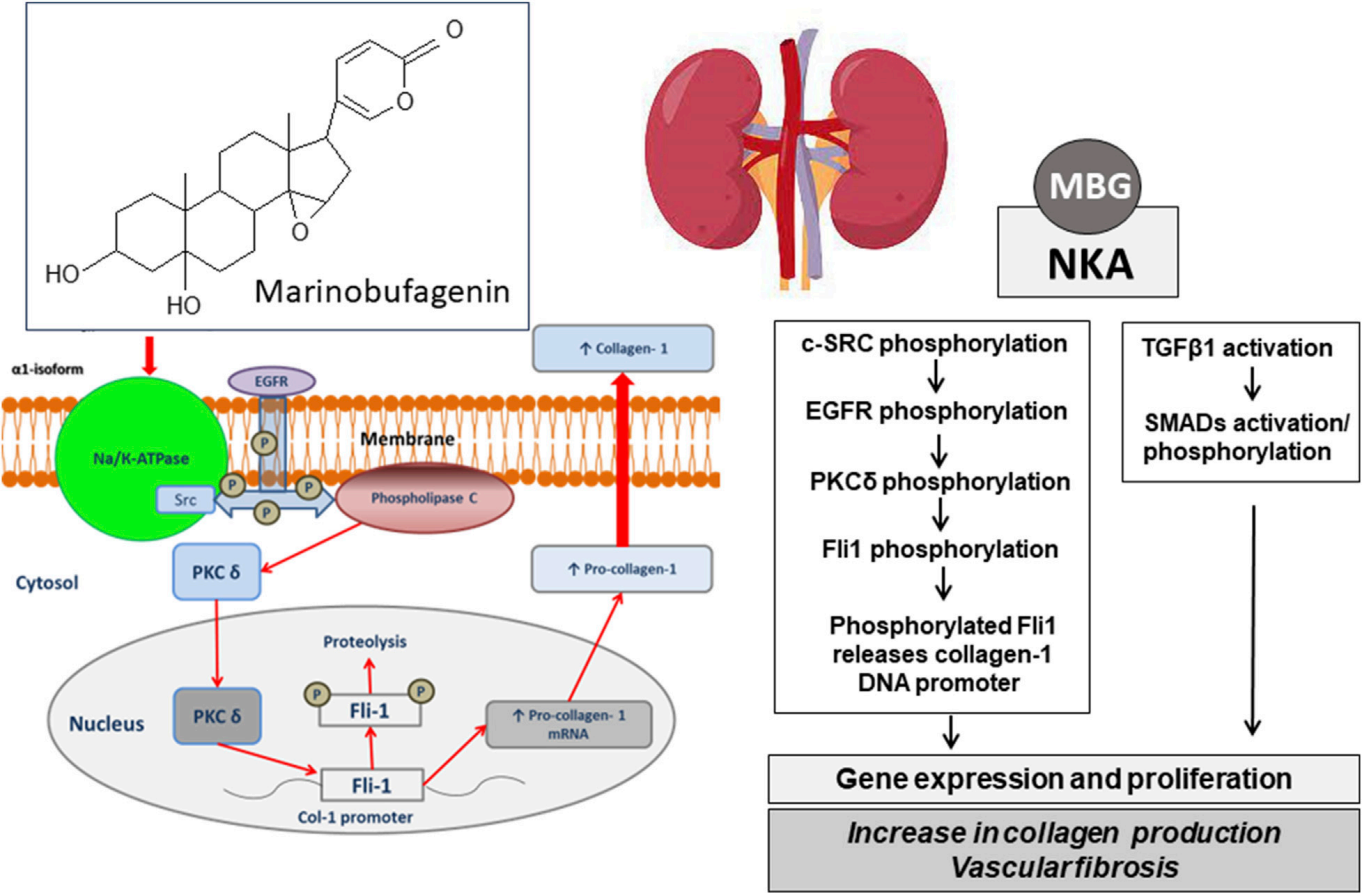
Chemical structre of bufadienolide, marinobufagenin and its pharmacological target, Na/K-ATPase. The schematic presentation of the possible molecular mechanisms of the implication of MBG in blood pressure regulation via ionic pathway (Na^+^/K^+^-mediated signaling) and stimulation of vascular fibrosis via Na/K-independent signaling. MBG, marinobufagenin; NKA, Na/K-ATPase; c-SRC, proto-oncogene tyrosine-protein kinase; EGFR, epidermal growth factor receptor; PLCγ, phospholipase C gamma; PKCδ, protein kinase C delta; Fli-1, Friend leukemia integration 1 transcription factor, a negative regulator of collagen-1 production; TGFβ, transforming growth factor beta; SMADs, Mothers Against DPP Homologs.

The inhibition of Fli1, a member of the erythroblast transformation specific (ETS) family, is critical for MBG-induced fibrosis (Elkareh et al., 2007). Fli1 acts as a negative regulator of collagen-1 synthesis and it competes with another transcription factor, ETS-1, to maintain a balance between stimulation and repression of the collagen-1 gene (Elkareh et al., 2007). The NKA/Src/EGFR complex begins a signal cascade, which activates phospholipase C resulting in the phosphorylation of PKCδ and its translocation to the nucleus. In the nucleus, phosphorylated PKCδ phosphorylates Fli1, which withdraws the Fli1-induced inhibition of the collagen-1 promoter and increases procollagen expression and collagen production (Elkareh et al., 2007; Nikitina et al., 2011; Haller et al., 2012; Agalakova et al., 2021). This mechanism of synthesis of collagen-1, Fli1-dependent fibrosis, emerges in several disorders associated with enhanced consumption of salt and includes age-dependent hypertension (Fedorova et al., 2023), preeclampsia (Nikitina et al., 2011), and CKD (Elkareh et al., 2007; Kolmakova et al., 2011). Pro-fibrotic effects initiated by MBG may also be TGFβ1/SMAD-dependent and underlie vascular fibrosis in salt-induced normotensive (Grigorova et al., 2018) and hypertensive rats (Zhang et al., 2019), but TGFβ sensitive mechanisms are not involved in preeclampsia (Nikitina et al., 2011) or chronic renal failure (Elkareh et al., 2007; Haller et al., 2012). These findings indicate the causative link between salt intake, vascular stiffness, and MBG, an endogenous natriuretic hormone and Na/K-ATPase inhibitor (Figure 1).

Over the past 20 years, the digoxin-specific Fab fragments (Digibind) have been successfully used in the treatment of poisoning by toad venom (Brubacher et al., 1999) and it has shown their effectiveness in animals with experimental hypertension (Huang et al., 2010). It is well known that in rats with renal failure (Haller et al., 2012) and experimental preeclampsia (Agalakova et al., 2022) antibodies to MBG or Digibind, antibodies that interact with CTS, are capable of reversal of hypertension and fibrosis of cardiovascular tissues. Several patients with preeclampsia have been successfully treated with Digibind over the last three decades (Adair et al., 1996; Adair et al., 2010; Lam et al., 2013). CKD is another example of a condition in which the role of CTS has been established, and *in vivo*, studies have demonstrated the contribution of CTS in the pathogenesis of left ventricular and renal fibrosis (Haller et al., 2012; Haller et al., 2014). However, the deleterious effects of MBG could be reversed via another way to antagonize the effects of CTS, by blockade of mineralocorticoid receptors by spironolactone.

## 4 MRA and Na/K-ATPase

**FIGURE 2:**
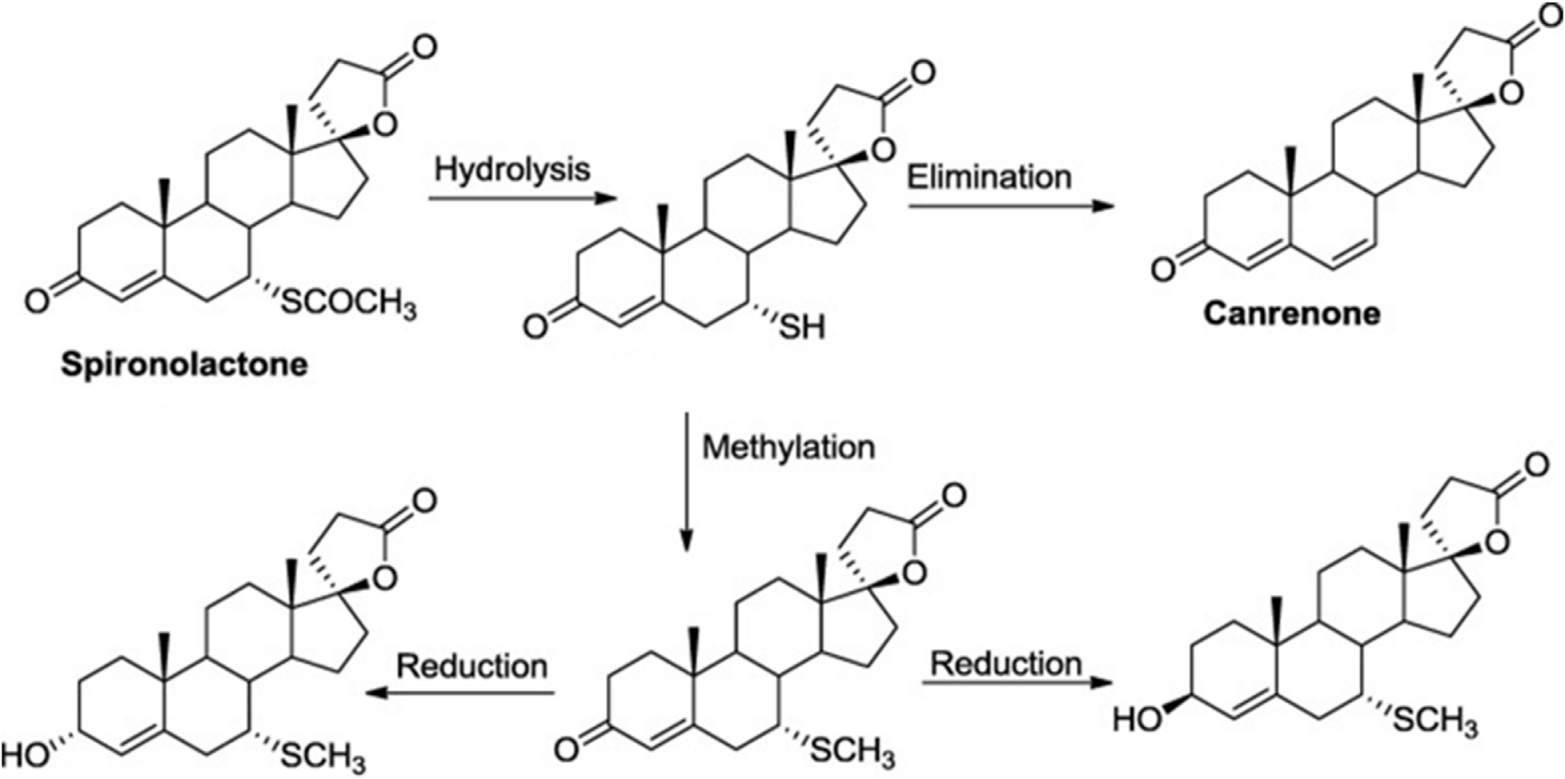
Schematic representation of the spironolactone metabolism in liver. Spironolactone has 100% bioavailability, *per os*, and after daily administration of 100 mg of spironolactone for 15 days its maximum concentration (C_max_) is 80 ng/mL. Binding with blood plasma proteins occurs at 98%. In the process of spironolactone biotransformation, active sulfur-containing metabolite 7-alpha-thyomethyl-spironolactone and canrenone are formed in the liver.

The idea that spironolactone or canrenone may function as a competitive inhibitor for cardiotonic steroids is not a novel concept. It all started in the late 1960s when Selye proposed the use of potassium-saving spironolactone diuretic to treat digitalis intoxication (Selye et al., 1969). Canrenone is the pharmacologically active metabolite of spironolactone, used in antihypertensive therapy (Figure 2). Canrenone exhibits its anti-aldosterone action because it blocks the binding of aldosterone to a cytosolic receptor in distal and collecting tubules of the nephron with subsequent inhibition of the synthesis of a specific protein that facilitates the entrance of Na + ions into the cell and the consequent increment of NKA (Sadee et al., 1973). Several studies suggest that canrenone interacts with the ouabain-sensitive NKA competitively, by antagonizing the binding of 3H-ouabain and MBG (Balzan et al., 2003; Tian et al., 2009). In several studies, canrenone was shown to act as an inhibitor of NKA (Belz and Kleeberg, 1973; Garay et al., 1985). Furthermore, if the pump was blocked by ouabain, canrenone could re-stimulate the pump (Semplicini et al., 1995). Thus, canrenone presents itself as a dual agonist/antagonist of the NKA (Finotti and Palatini, 1981).

Schreiber et al. (1981) demonstrated the presence of a digoxin-like immunoreactive substance in the serum of rats with cardiac overload. In uremic patients a circulating factor was described, whose activity is diminished by hemodialysis it was suggested to be an important endogenous regulator of the NKA (Izumo et al., 1984). In 1983 a group from Germany showed that in uremic rats diminished sarcolemmal NKA activity in the heart may be related to increased levels of natriuretic factor, i.e., endogenous digitalis-like substances (Kreusser et al., 1983). Shortly thereafter a digitalis-like factor that was different from ouabain and digoxin was isolated from the peritoneal dialysate of hypertensive patients with kidney failure (Graves et al., 1993). In 1988 using polyclonal antibody against MBG we demonstrated that MBG-like CTS exhibits increases in patients with CKD (Gonick et al., 1998), and several years later this observation was confirmed using a monoclonal antibody, while another CTS, endogenous ouabain, did not become elevated (Kolmakova et al., 2011). This observation of elevated MBG levels in experimental animals and patients with CKD was confirmed by the other groups of investigators (Paczula et al., 2019; Bolignano et al., 2023a; Bolignano et al., 2023b; Carullo et al., 2023).

Spironolactone and its active metabolite, canrenone, were reported to lower blood pressure in rat hypertension models, in which levels of CTS are elevated (Grichois et al., 1986; Pamnani et al., 1990). In hypertensive patients, a low dose of aldosterone antagonist added to antihypertensive treatment significantly improved left ventricular diastolic function (Grandi et al., 2002). Finotti and Palatini (1981) suggested that that canrenone interacted with isolated NKA at the same site as digitalis. Garay et al. (1986) demonstrated that canrenone attenuated digitalis-induced inhibition of the NKA in human red blood cells whereas Balzan et al. (2003) made similar observations in human red blood cells and placenta. Sorrentino et al. (1996) observed that canrenone could antagonize the vasoconstrictor effects of ouabain. In the study of a group of hypertensive patients, Boero et al. (1989) demonstrated a profound inhibition of erythrocyte Na/K-pump activity after saline infusion which was reversed by canrenone. Plasma from hypertensive patients obtained before saline infusion significantly inhibited the NKA of erythrocytes from normal subjects, and vice versa, while plasma taken after the saline infusion plus canrenone was unable to produce any significant inhibition (Boero et al., 1989). Two years later, Finotti and Palatini (1981) developed a theory of partial agonism/antagonism of canrenone against NKA depending on the conformation of the enzyme and the combined effects of complete agonists or inhibitors. Summarizing data on endogenous substances that inhibit the cell membrane Na/K-pump in the renal tubules and reduce sodium reabsorption in which canrenone exerts both agonist and antagonist effects on the digitalis receptor site of the NKA, Semplicini et al. (1995) suggested that this substance may belong to a new class of compounds, antagonists of endogenous digitalis-like factor, i.e., CTS. Aldosterone antagonists are effective antihypertensive agents in animal models of hypertension and in patients with essential hypertension. Administration of canrenone for example, reduced blood pressure, increased red blood cell NKA activity, and antagonized vasoconstriction (Semplicini et al., 1995).

## 5 Mineralocorticoid antagonists in CKD

Mineralocorticoid receptor antagonists (MRA) are known to reverse cardiovascular fibrosis (Buffolo et al., 2022; Verma et al., 2024). Because MBG induces fibrosis through the Fli1-dependent mechanism we studied the effects of spironolactone and its main metabolite, canrenone, on fibrosis in a series of experiments (Elkareh et al., 2009; Tian et al., 2009; Nikitina et al., 2011; Haller et al., 2012). In subtotal 5/6 nephrecomized rats, it has been demonstrated that both spironolactone and canrenone impair MBG-induced increase in collagen synthesis and inhibit tritiated ouabain binding (Tian et al., 2009). Recently, it has been demonstrated that nanomolar concentrations of MBG stimulate the synthesis of collagen and induce fibrosis in cardiovascular tissues and in the kidney (Elkareh et al., 2009). *In vivo*, the administration of MBG in a concentration that is observed in renal failure caused the development of cardiac fibrosis with activation of the signaling pathway mediated by NKA, which was confirmed by an increase in Src and phosphorylation of a mitogen-activated protein kinase (MAPK) in the myocardium (Elkareh et al., 2009). Most recently spironolactone was reported to suppress cardiac fibrosis in rats chronically treated by MBG (Lam et al., 2013). Notably, in this study, MBG exhibited a pro-fibrotic effect in the absence of changes in aldosterone levels (Lam et al., 2013). Importantly, in rats with pregnancy-induced hypertension high levels of MBG were associated with high blood pressure, stiffening of umbilical vessels, and elevated vascular level collagen-1, and *in vitro* incubation of the healthy blood vessels in the presence of low MBG concentration produced similar phenotype (Elkareh et al., 2007). In healthy rats, it was shown that aldosterone antagonists can also reverse MBG-induced vascular fibrosis, in the explants of the thoracic aorta and the cultured rat vascular smooth muscle cells, and we observed that canrenone suppressed the effect of MBG synthesis of collagen-1 (Fedorova et al., 2015). This observation was confirmed by clinical data which showed that in patients with resistant hypertension receiving spironolactone as an addition to the conventional antihypertensive therapy, there was a decrease in aortic vascular stiffness in parallel with an increase in the erythrocyte NKA (Fedorova et al., 2015). It has been established that MRA decrease blood pressure and suppress cardiac fibrosis in rat models of renal failure in which levels of CTS including MBG are elevated (Elkareh et al., 2009; Haller et al., 2012). Importantly, CTS-induced vascular fibrosis may not be associated with hypertension but rather be accompanied by impaired vascular relaxation, for example, in NaCl-loaded rats with diabetes mellitus (Fedorova et al., 2019) and in normotensive non-dippers from the African-PREDICT study (Strauss-Kruger et al., 2020). Thus, an interaction between NKA and MBG could be a target for aldosterone antagonists.

The last two decades were associated with enhanced interest in to use of MRA in CKD, because 1) several clinical trials found the effects of aldosterone antagonists on blood pressure promising, and 2) the absence of results demonstrating that aldosterone antagonists cause serious hyperkalemia (Georgianos and Agarwal, 2023; Tuttle et al., 2024; Yuan et al., 2024). These facts raised a question of whether in CKD patients receiving hemodialysis, spironolactone treatment would lead to significant changes in PVW left compared with placebo. First, in 2001 in a group of 153 patients receiving hemodialysis was studied, the blood pressure decreased along with pulse-wave velocity (PWV) (London et al., 2001). Next, when a group of patients with early-stage CVD treated with 25 mg spironolactone was studied they found that PWV went down and a decrease in the levels of pro-peptide of type III procollagen was found (Edwards et al., 2009; Edwards et al., 2010). Subsequently, Boesby et al. (2013) studied patients with CKD and found the effect of eplerenone in CKD stages 3–4, but they did not see a significant reduction of pulse pressure velocity but observed a significant decrease in the augmentation index. In our study in a small group of patients with resistant hypertension and mild CKD (70 mL/min) receiving spironolactone as an addition to the antihypertensive therapy there was a decrease of aortic stiffness in parallel with an increase in the erythrocyte NKA (Fedorova et al., 2015). Finally, Eklund et al. (2022) found no evidence supporting an effect of 12-week administration of spironolactone 50 mg daily on vascular stiffness, cardiac systolic, or diastolic function in hemodialysis patients. Interestingly, the same mechanism of synthesis of collagen-1, Fli1-dependent fibrosis, emerges in several disorders associated with enhanced consumption of salt and includes CKD (Haller et al., 2012). Excessive dietary NaCl may also alter vascular structure and function via cardiotonic steroid mechanisms in age-associated reductions in renal blood flow and in the ability to excrete sodium.

Importantly, spironolactone increased the incidence of the moderate hyperkalemia but not of severe hyperkalemia, and these findings demonstrated that spironolactone could be used safely in patients on hemodialysis even in the lack of cardiovascular events (Charytan et al., 2019; Patel et al., 2022). Another study showed that treatment of CKD patients receiving hemodialysis with 25 mg of spironolactone daily for 3 years reduced the incidence of cardiovascular and cerebrovascular mortality or hospitalization compared with placebo, all-cause mortality was also reduced by 60% in the spironolactone-treated group compared with controls (Matsumoto et al., 2014). Several studies demonstrate that finerenone, a non-steroidal mineralocorticoid receptor antagonist, improves cardiorenal outcomes in patients with CKD and type 2 diabetes with a manageable hyperkalemia risk and a reduction in hypokalemia (Bakris et al., 2019; Ruilope et al., 2019). Accordingly, the most recent guidelines for CKD indicate that nonsteroidal MRA is most appropriate for the treatment of adults with type 2 diabetes who are at high risk of CKD progression (Kidney Diseases: Improving Global Outcomes. KDIGO, 2024). Novel biomarkers, including amino-terminal type III procollagen peptide (PIIINP), PICP, FGF23, marinobufagenin, and several miRNAs, show promise for early detection and risk stratification.

## 6 Marinobufagenin and preeclampsia

Preeclampsia is another example of the condition associated with the activation of CTS and Fli1-dependent pro-fibrotic signaling. Preeclampsia is associated with a high plasma MBG level, a four-fold decrease in Fli1 level, and a three-fold increase in collagen-1 level in the umbilical arteries versus those from normal subjects (Agalakova et al., 2022). Isolated rings of umbilical arteries from the subjects with preeclampsia exhibited impaired responses to the relaxant effect of sodium nitroprusside as compared to control vessels. The effects of preeclampsia on Fli1 and collagen-1 were blocked by the *in vitro* treatment of umbilical arteries with 10 mol/L canrenone (Agalakova et al., 2022). Remarkably, when healthy umbilical arteries are pretreated with MBG they acquire properties of preeclamptic vessels, and they become stiff and fibrotic (Agalakova et al., 2022). These data demonstrate that elevated MBG level is implicated in the development of the fibrosis of umbilical arteries in preeclampsia and that this could be blocked by MRA. It was noted that when pregnant rats were treated with 40 mg spironolactone from 13 to 21 days of pregnancy, male fetuses showed signs of feminization (Hecker et al., 1980). Therefore, spironolactone was not advised for humans during pregnancy (Regitz-Zagrosek et al., 2011) but considering that eplerenone has not been associated with adverse effects during pregnancy in animal studies, this drug is likely to be a better choice for use in pregnant women than spironolactone.

## 7 MRA and liver fibrosis

Interestingly, most recently in several species (mice, rats, and humans), MRA made a promise as a pharmacological treatment for alcohol addiction (Farokhnia et al., 2022). The mechanism of action by which spironolactone reduces alcohol consumption is an area of investigation, but the authors hypothesize that increased levels of circulating aldosterone may contribute to alcohol drinking by increasing anxiety, facilitating brain stress system activation, and/or inducing neuroinflammation (Farokhnia et al., 2022). We confirmed these results and demonstrated that interaction between Na/K-ATPase and MBG could be a target for aldosterone antagonists, and that MRA reversed MBG-induced elevation of blood pressure associated with voluntary ethanol intake (Kashkin et al., 2018). Results of another experiment show that spironolactone is reducing the reinforcing effect of ethanol by modulation of NKA activity or/and by competitive interaction with MBG (Kashkin et al., 2013).

These data suggest that spironolactone treatment has a new perspective on the therapy of alcohol abuse. Indeed, in a recent review (Diaz et al., 2023) activation of the immune system under the effect of ethanol can be triggered by pathogen-associated molecular patterns, cytokines, which, in turn, promote liver inflammation and the progression of liver fibrosis (Diaz et al., 2023). Potential therapeutic targets for the treatment of liver fibrosis include the antagonist of the mineralocorticoid receptor system, spironolactone (Diaz et al., 2023). Interestingly, as early as 1949 Rein suggested that endogenous strophanthin factor, i.e., CTS, with a lack of oxygen, is liberated from the spleen/liver unit. This substance, “hypoxia-lienin” shows in its effects a wide range of similarities with the strophanthin. This paper by Rein (1949) is especially interesting because the later results demonstrate that steroid MBG, is very likely to be synthesized via a bile acid pathway i.e., it could come from the organ extremely rich with bile acids, the liver (Fedorova et al., 2015). Importantly all the specific chemical reactions in the transformation of bile acids into bufadienolide molecules have been described in amphibians (Fedorova et al., 2015).

## 8 Future perspectives

Spironolactone and canrenone have been used in medical practice since the sixties first as a calcium-sparring diuretic (Feldman, 1975) then as an integral part of the treatment of left ventricular failure (London et al., 2001). Finally, the latest treatment guidelines state that nonsteroidal MRAs are indicated in the treatment of CKD in adult patients with type 2 diabetes. Above described three examples from the areas in which aldosterone receptor antagonists are studied, CKD, preeclampsia, and alcohol use disorder demonstrate the repositioning of spironolactone and other antagonists of mineralocorticoid receptors. The mechanism of myofibroblast activation is the central issue in kidney fibrosis. Myofibroblast activation and subsequent ECM accumulation are major events in kidney fibrosis. The activated myofibroblast is the prominent contributor to renal fibrosis due to its ability to produce the most matrix (Yuan et al., 2019). There are several sources of profibrotic factors and key signals that mediate myofibroblast activation, growth factors, chemokines, cytokines, and bufadienolides including MBG. Unfortunately, no effective drugs at present exist against kidney fibrosis. However, several drugs can only delay the progression of CKD: renin-angiotensin system blockers, SGLT2 inhibitors, GLP-1 receptor agonists, endothelin-1 blockers or non-steroidal MRAs. In addition to renal protection MRAs known to reverse CV fibrosis, a very promising new treatment for uremic cardiomyoptahy. There is also a lack of good biomarkers to predict and assess kidney or cardiac fibrosis in clinical practice. Therefore, a better understanding of the pathogenesis of kidney fibrosis, and access to drugs stopping or even reversing it, like anti-MBG monoclonal antibody which causes a decrease of collagen-1 in aorta of rats with CKD (Agalakova et al., 2024). These findings indicated the causative link between vascular stiffness, and CTS and suggest that interaction between NKA and MBG could be a target for aldosterone antagonists. A novel treatment of CKD already involves the use of mineralocorticoid receptor antagonists capable of impairing NKA interactions with its endogenous ligands while preeclampsia and alcohol use disorder are still under the development.
